# Turning the Page on Hardcopy Risk Management Plan Educational Materials: Digitalization Made Possible

**DOI:** 10.1007/s43441-024-00717-3

**Published:** 2024-11-25

**Authors:** Anabel Ng, Ayn Nova Celo, Beliz Fernandes

**Affiliations:** 1 Novartis Asia Pacific Pharmaceuticals Pte Ltd, 20 Pasir Panjang Road, #10-25/28, Mapletree Business City (West Tower), 117439 Singapore; 2 Novartis Healthcare Private Limited, Inspire BKC, 7th Floor, Bandra Kurla Complex, Bandra (East), 400 051 Mumbai, India

**Keywords:** e-RMP, Risk management plan, Digitalization, Electronic education materials

## Abstract

‘Risk Management Plan Educational Materials’ (RMP EMs) are additional risk minimization measures (aRMMs) intended to prevent or reduce the occurrence of adverse reactions associated with the exposure to a medicine, or to reduce their severity or impact on the patient. While the healthcare sector is embracing various digital tools and platforms for educational and/or awareness building purposes, paper-based materials have remained the mainstay approach for implementation of aRMMs by pharmaceutical companies. Novartis in Singapore conducted a pilot on the feasibility of distributing electronic copies of RMP EMs (e-RMP). Post-pilot, e-RMP was officially implemented in Novartis Singapore. A year following the launch of e-RMP, a survey was performed with healthcare professionals (HCPs) to understand end-users’ experience. The survey responses revealed a general preference by both HCPs and patients towards e-RMP. Digital methods of delivering educational aRMMs offer great benefits over traditional paper-based programs. e-RMP significantly reduces the time needed for updated RMP EMs to reach HCPs and their patients/caregivers. This is important to ensure that HCPs and patients/caregivers are made aware of any updates in key safety messages of the products in a timely manner to ultimately ensure patient safety. The successful transition to digital solutions requires purposeful collaborations between key stakeholders of the healthcare ecosystem including regulatory authorities, pharmaceutical companies, HCPs, patients and caregivers. This article aims to provide insights on the digitalization journey of e-RMP, a case study in Singapore, outlining the value but also some of the challenges faced during this transformation.

## Introduction

Risk Minimization Measures (RMMs) are interventions intended to prevent or reduce the occurrence of adverse reactions associated with the exposure to a medicine, or to reduce their severity or impact on the patient should adverse reactions occur [1]. While routine RMMs such as product labelling may be sufficient for most medicinal products, some medicines with specific safety concerns require implementation of additional RMMs (aRMMs) [2]. aRMMs include Educational Materials (EMs) for Healthcare Professionals (HCPs) and/or patients/caregivers, pregnancy prevention programs and controlled access programs [1].

Product-specific Risk Management Plan (RMP) EMs contain ‘key safety messages’ to inform HCPs and/or patients/caregivers about the safety risks associated with the medicines being consumed, with the intention to ultimately protect the safety of patients. RMP EMs ensure that the HCPs and/or patients/caregivers are aware of the safety risks associated with the medicine and how to either prevent certain anticipated safety events, or how to detect signs and symptoms early for quick and effective management [1, 2].

Risk management is an ongoing activity where new safety information about the medicinal products may be identified pre- or post-authorization. The safety profile of the medicinal product can evolve throughout the product’s life cycle, which may warrant the need for additional or new RMMs. Alternatively, it may require updating the messages conveyed in a particular aRMM such as EMs [3]. The EMs must be continuously implemented and maintained as long as they remain as a commitment in the effective RMP. This is because the EMs are assessed to be necessary in maintaining the risk-benefit balance of the product [2]. This means that the EMs for a particular product must be continuously distributed to any new HCPs and new patients/caregivers (via their HCPs). In addition, as per the authors’ experience, the frequency of updates to an EM varies, but can be as often as once to twice a year for some products due to changes in their safety profile. With each round of revisions, the updated EMs must be distributed to all relevant prescribers and patients/caregivers (via their HCPs). As most EMs are traditionally implemented as hardcopy, this presents multiple challenges that Market Authorization Holders (MAHs) may face such as strain on resources to print and distribute new/updated EMs [4], and potential delays in EMs reaching the target audience as time is needed to deliver the materials to the various clinics and institutions, and where applicable, to the patients/caregivers through their HCPs. Such delays would become even more pronounced when pharmaceutical companies’ access to healthcare institutions are impacted, be it temporarily due to crises such as the COVID-19 pandemic, or progressively as more institutions deny commercial visits [5, 6].

While the COVID-19 pandemic posed severe challenges to healthcare systems and governments in many countries, it also accelerated the uptake of electronic solutions worldwide. Consequently, the use of Quick Response (QR) codes became more widespread, with many individuals becoming familiar with their functionality [7]. This creates many opportunities for the pharmaceutical industry, leveraging on the increased Information Technology (IT) literacy among consumers to drive process changes. While digitalization in the pharmaceutical industry may not be a new concept [8], there are limited Health Authorities’ (HAs) guidances regarding the digitalization of RMP. With the anticipated update to the European Medicines Agency (EMA) Good Vigilance Practice (GvP) Module XVI (EMA/204715/2012 Rev 3* - Draft for public consultation), there appears to be a slight shift towards electronic dissemination of EMs. A study performed by Tillmann et al. also demonstrates interest from HCPs and patient stakeholders in digital approaches to educational aRMMs [4].

This article aims to share the experience from Novartis Singapore, on its journey to digitalize RMP EMs and their distribution to HCPs and patients/caregivers.

### Case Study in Singapore

The Singapore HA, Health Sciences Authority (HSA), may request aRMMs to be implemented for certain products, at either the pre- or post-marketing stages, due to identified significant safety concerns related to the product [9]. Consequently, MAHs in Singapore must ensure that all relevant HCPs are provided a copy of the latest HSA-approved materials for their knowledge (for HCP EMs), or for further dissemination to their patients/caregivers (for patient EMs). Nonetheless, the guidance does not specify the format of the EMs [9]. It also does not specify any additional requirements, such as the type of evidence to be collected, should the MAH decide to adopt the implementation of EMs in electronic formats.

The most common approach to implement RMP EMs in Singapore was to print and distribute hardcopy EMs via in-person visits or postage mails [10]. However, in a survey conducted by HSA to understand prescribers’ experiences with RMP EMs and their preferences towards receipt of such EMs, it was revealed that the least preferred distribution channel was hardcopy EMs provided by mail. Instead, majority of the respondents (64%) preferred receiving digital copies of EMs [10]. This indicates that there is an opportunity to transform the RMP EM implementation process, potentially enhancing communication of important safety messages while minimizing the company’s carbon footprint. In addition, with the trial and official implementation of electronic labelling (e-labelling) in Singapore since 2019 and 2021 respectively, HCPs are already accustomed to digital alternatives [11, 12]. To Novartis Singapore, a logical progression in our digital transformation would be to transition beyond product labels for HCPs, moving to RMP EMs for both HCPs and patients.

## Methods

### e-RMP Mechanics and Alignment with Local Health Authority

Novartis in Singapore developed a strategy to distribute hardcopy cards containing a QR code (QR code cards) to HCPs, and to patients/caregivers via their treating physicians during their visit to the clinic/institution. The QR code cards act as a vehicle, directing users to the webpage hosting electronic copies of the RMP EMs (e-RMP) when they scan the QR code. The webpage would include (i) Package Insert(s), (ii) Educational Material(s) for patients, if applicable, and (iii) Educational Material(s) for HCPs, if applicable.

The hardcopy QR code cards would also contain the product name, the type of material e.g. Patient Booklet or Physician EM, an alternative Uniform Resource Locator (URL) link to the material(s), and a reminder applicable to patient EMs for patients/caregivers to consult their healthcare team in case of questions on the treatment or regarding the EMs.

As and when the EMs are updated, they would be uploaded onto the same webpage, superseding the outdated material. Novartis Singapore will then notify HCPs via email regarding the updated EMs. The HCPs can access the updated e-RMP by scanning the QR code on the cards that were previously distributed to them. However, this notification via email can only be sent to HCPs who have provided consent to receiving such communications from Novartis. Therefore, the QR code cards will be distributed again only to the HCPs who did not provide consent, as a way for the company to notify them on the updated EMs. Patients/caregivers would in turn be notified of the updated EMs by their HCPs during their next visit. If the patient/caregiver has misplaced the QR code card that was previously provided to them, their HCP will provide them with another copy of the QR code card.

Although there is an existing and currently effective HSA guidance regarding implementation of e-labelling, the current HSA guidance on RMP EMs had no specifications for e-RMP [9]. Furthermore, there was no known local industry practice to implement digital EMs at the point of conceptualization. Therefore, Novartis proactively engaged HSA, notifying HSA regarding the plan to implement e-RMP, and incorporated inputs and suggestions from HSA with regards to the implementation of digital EMs.

Novartis ensured that the website hosting the EMs adhered to the requirements prescribed under the Health Products Act (HPA) and the Health Products (Advertisement of Specified Health Products) Regulations, where promotion or advertisement of the use of prescription-only medicines is prohibited [13].

In the interest of less tech-savvy patients/caregivers who may not have internet access, or due to other factors, may not be able to obtain the materials online, an opt-in system was implemented. EMs were implemented as e-RMP by default, but hardcopy EMs were made available upon HCPs’ requests (on behalf of their patients/caregivers). This will ensure that this group of patients/caregivers are still adequately made aware of the safety concerns associated with the medicines that they are taking. This is also in line with insights received during an advisory board meeting with four patient associations in Portugal. They highlighted that there is still variability among patients/caregivers regarding their preferences towards paper or digital EMs, and that EMs should be made available in both formats, allowing patients/caregivers to choose the one that is more convenient for them [14].

### Pilot and Approach for Official Launch of e-RMP

The e-RMP pilot was implemented from 01 May 2021 to 30 Nov 2021. At that time, there were seven company products with RMP EMs in Singapore. Three of the products were not included in the pilot due to the nature of how the EM is to be used by the target audience, as well as organizational complexities and priorities during that period. Four Novartis products across different therapeutic areas including Neuroscience, Ophthalmology and Hematology were included in the pilot, with a total of 18 EMs– three for HCPs and 15 for patients/caregivers. Three of the four products in scope had EMs to be updated due to updates to the EU RMP within the period of the pilot, while the EM for the remaining product was redistributed as e-RMP solely for the purpose of the pilot, with no change in scientific content from the previous version. The e-RMPs were distributed to all the HCPs who were identified as prescribers of the products in scope. As a result, a total of 224 HCPs received the QR code cards as part of the pilot.

A communication letter was provided to all HCPs prescribing these four products, alongside the initial round of distribution of the QR code cards. This was done to inform the HCPs of the change in the format of EMs distributed as part of this pilot and to provide them with contact details should they require any clarification or wish to provide any feedback.

At the end of the pilot, various considerations were taken into account to determine if e-RMP should be implemented as part of the company’s official process, namely feedback from HCPs and their patients/caregivers during the pilot, level of digital literacy amongst HCPs and/or patients/caregivers, and nature of the EMs.

The company decided to adopt a stepwise and tailored approach towards e-RMP– full hardcopy EMs, hybrid e-RMP and full e-RMP. Table 1 shows the differences between the three approaches. An opt-in system was retained for products implemented as full e-RMP, whereby HCPs and/or patients/caregivers who prefer paper-based EMs could still receive them upon request. The hybrid e-RMP approach would be that different EMs of a single product will be implemented differently (e.g. patient EM as hardcopy and HCP EM as e-RMP). With the long-term goal of implementing all products’ EMs as e-RMP, hardcopy EMs under the hybrid approach would still include a QR code and alternative URL printed on the EM. This would prompt users to refer to the electronic EM hosted online, which is added with the intention to familiarize users and improve eventual receptiveness towards e-RMP.

EMs for each product were thus analyzed individually, assessing the patient/caregiver population and type of EM, to determine which approach was most suitable. For example, if the EMs for a product include materials for both patients and HCPs, but there were concerns regarding the patient/caregiver population’s digital literacy to navigate to the e-RMP, a hybrid e-RMP approach may be more suitable. Similarly, if the EMs are actively used by the patients/caregivers to track certain information such as medication adherence (e.g. patient diary), it may be more appropriate to keep the EMs as hardcopy, until the necessary interactive functionalities can be incorporated into digital EMs.

**Table 1:** Summary of RMP EM approaches

	Hardcopy EMs	Hybrid e-RMP	Full e-RMP
**Default format of RMP EMs**	Hardcopy EMs	Different for different EMs of the same product (e.g. QR code card for HCP EMs and hardcopy* for patient EMs) * QR code and URL to be added to the material to direct users to the website hosting the electronic RMP EMs if they would like to refer to an electronic copy instead	Hardcopy QR code card
**Upon request (opt in)**	Not applicable	Hardcopy EMs	

When preparing for the official launch of e-RMP, a decision was made by Novartis Singapore to change the website hosting the EMs. Prior to the roll out of e-RMP, this website was targeted towards HCPs only, and was used to host both promotional and non-promotional materials. The website required HCPs to register an account and log in before they could view the contents. However, as e-RMP can be for HCPs and/or patients/caregivers, there was a need to ensure that the webpage hosting e-RMP was freely accessible to the public, without the need for logging-in which may deter users from viewing the materials. At the same time, care had to be taken to comply with the Health Products (Advertisement of Specified Health Products) Regulations. Therefore, a new e-RMP webpage was created within the current website, where only the e-RMP webpage and its contents remain publicly accessible. This differentiates the e-RMP webpage from other webpages within that website that host non-RMP contents (promotional and/or non-promotional), which are access-controlled pages that are restricted to registered users (HCPs) only. As a result, all target audience of the e-RMP (HCPs and/or patients/caregivers) can freely access the EMs, while control of the access to other types of resources for HCPs are still maintained, all within a single website.

### Effectiveness Check

According to Mouchantaf, R. et al., there is currently no single established consensus on the method for assessing the effectiveness of RMMs [15]. Some ways to evaluate the success of RMP EMs could include knowledge assessment, changes in clinician behaviors, or improvement in clinical outcomes, such as a reduction in the frequency or severity of the adverse reactions related to the safety concerns communicated in the EMs [1, 16]. Since e-RMP is the change in the approach of RMP EM implementation (from hardcopy to digital), rather than changing the scientific content of the EMs which may occur regardless of whether the EM is implemented as e-RMP or hardcopy, effectiveness checks to evaluate the success of e-RMP in conveying and educating the users on the safety concerns was considered out of scope of this project.

Through e-RMP, there was a transition in the format and the way the EMs were delivered. Therefore, similar to publications researching on digital aRMMs [4], an effort was made to collect feedback from the HCPs and their patients/caregivers through a survey with HCPs in 2023, a year after the official implementation of e-RMP. The aim of the survey was to understand whether e-RMP has resulted in improved accessibility and usage of the RMP EMs, and whether this is the preferred distribution channel for HCPs and their patients/caregivers. It consisted of five questions. In addition to the questions listed under Table 2, there was also a question to identify which product the HCP prescribes, and a final question that was open-ended, inviting feedback from HCPs or their patients about e-RMP. Considering that government hospitals enforce a firewall which prevents HCPs from completing online surveys, the approach adopted for the survey was for company representatives to engage as many HCPs in-person during their routine field visits to go through the survey questions. The survey ran for four months, and participation was voluntary. By the end of the survey period, due to limited time to interact with HCPs during routine field visits and other competing agendas such as discussion of scientific articles as requested by the HCPs or sharing of updates to reimbursements in relation to the products, only around 20% of the total HCPs who received QR code cards after the official launch of e-RMP could be approached to complete the survey.

## Results

### Pilot

There was 100% uptake of e-RMP during the pilot and no objections were raised by the 224 HCPs who received the QR code cards, and their patients/caregivers. There were no requests for hardcopy materials for all HCP EMs, while only 5 HCPs (2.2%) requested physical copies of the patient EMs for one of the four products. This highlights the general receptiveness of the HCPs and patients/caregivers towards referring to electronic EMs. HCPs’ inclination towards digital EMs observed in our pilot was largely in line with the results from the survey conducted by HSA in 2018 to 2019 where majority of the respondents preferred receiving digital copies of EMs [10].

e-RMP can significantly reduce the time it takes for updated EMs to reach HCPs and patients/caregivers, which is essential in enabling patients’ safety. Based on the experience of the authors’ company representatives, the estimated time required for the distribution of conventional hardcopy EMs to HCPs, including travelling to the institution and waiting in the clinic to meet with the HCP, is about 40 min per doctor for each unique institution. In addition, distribution of the updated hardcopy EMs to patients/caregivers may be delayed as they only receive the EMs during their next scheduled appointment. Conversely, e-RMP enables electronic distribution and notification such as sending an email to inform HCPs that the updated EM has been uploaded, which only requires an estimated one minute per HCP. This shows an astonishing 97.5% reduction in the time needed for the EM distribution step. This reduction may be even more significant in larger countries where the travelling time is more substantial, or where the barriers to face-to-face distribution are higher. The updated e-RMPs are also available to the patients/caregivers immediately after the uploaded EMs have been replaced, without the need for waiting till their next doctor’s appointment.

Moreover, the above estimate does not include the time saved from the preparation prior to distribution and notification. Including the time required for the preparatory steps would result in a further reduction of approximately one week, which is the usual turnaround time by printing vendors engaged by the authors’ company, as the notification only requires an hour to draft, obtain approval and send out. Conversely, it can take up to a week to complete the liaising with the vendor and printing of the hardcopy EMs.

e-RMP effectively eliminates the need to print updated EMs, as the QR code cards remain valid regardless of any updates to the materials. Users would always have access to the latest materials that are hosted online. Estimated cost savings from the pilot amounted to USD$13,400 as the company did not need to print the updated EMs for the four products. This was based on the number of copies of EMs that would have been printed for the four products if they were implemented as traditional hardcopy EMs. This includes 18 EMs, where five were audio materials. The cost savings would be even more significant for companies with a larger number of audio EMs.

### Effectiveness Check

**Fig. 1:**
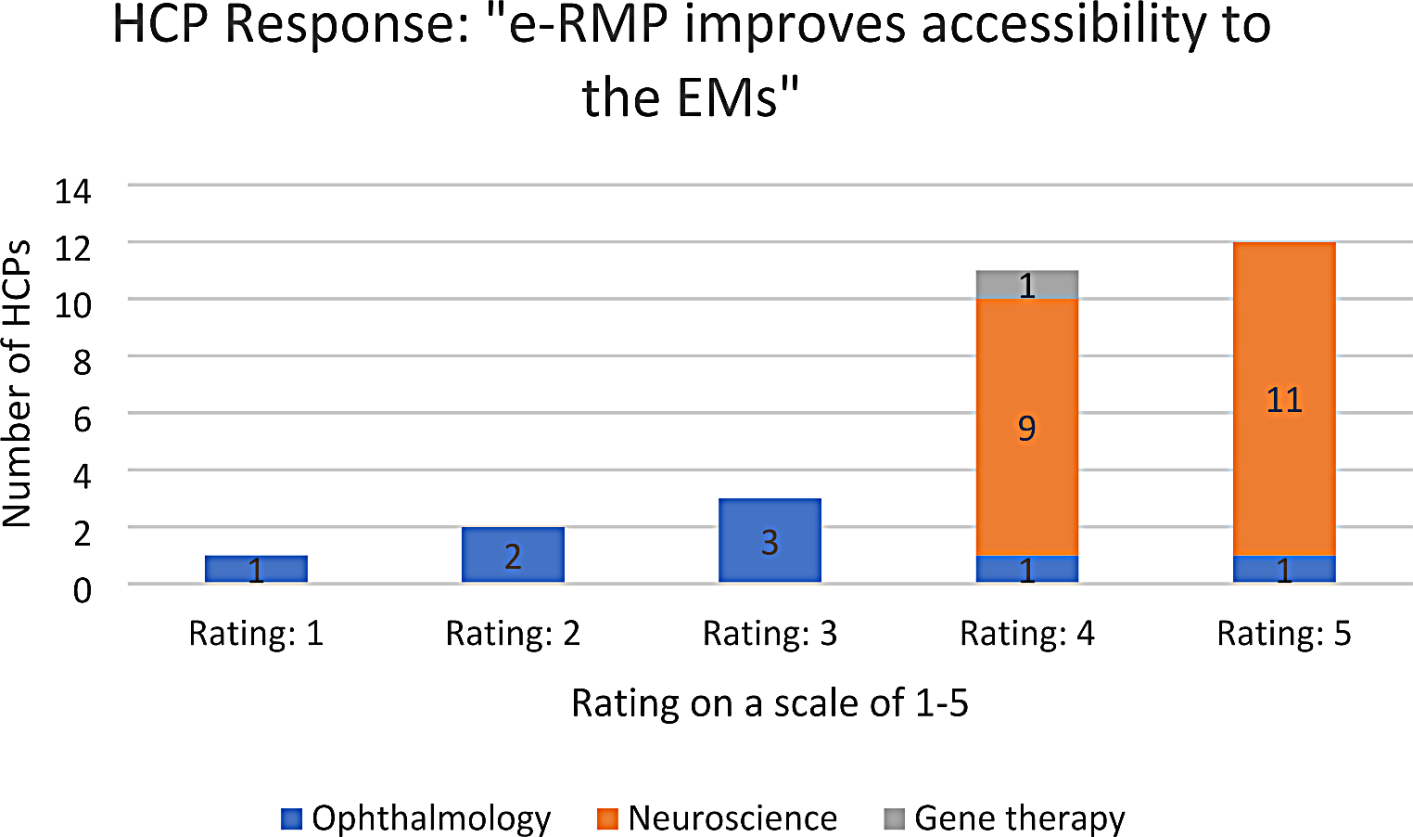
Breakdown of responses for survey question 1 (1 = completely disagree; 5 = completely agree)

**Fig. 2:**
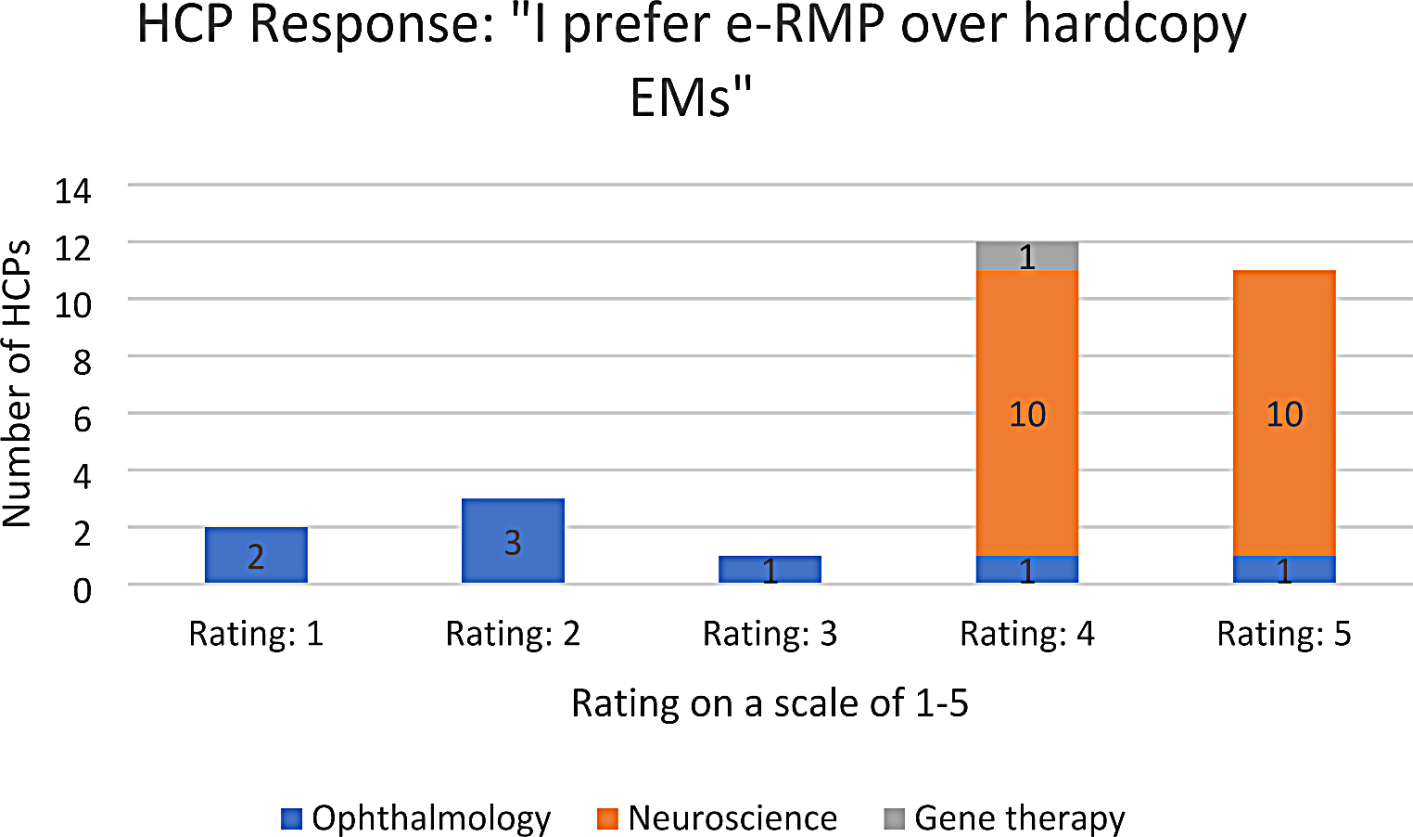
Breakdown of responses for survey question 2 (1 = completely disagree; 5 = completely agree)

**Fig. 3:**
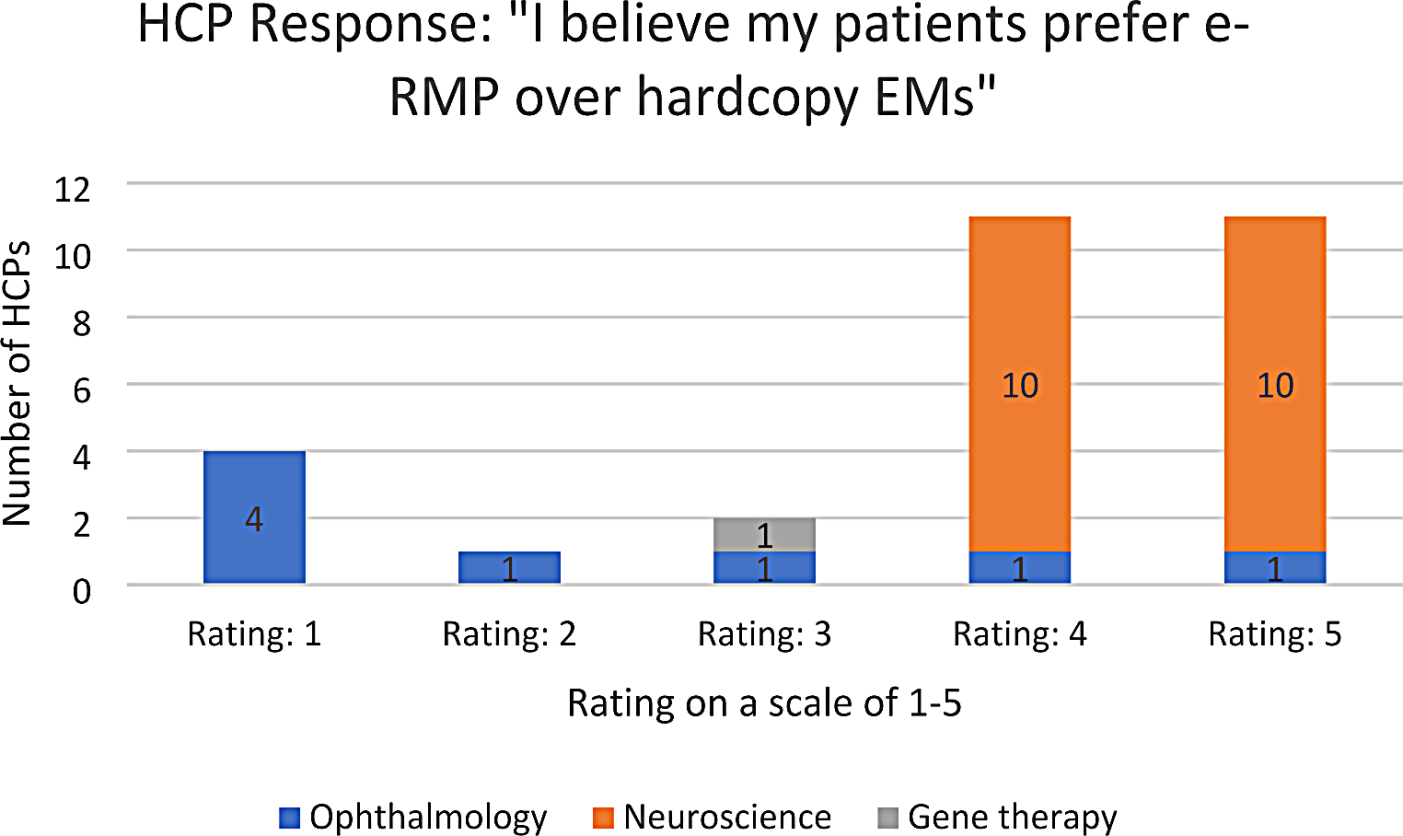
Breakdown of responses for survey question 3 (1 = completely disagree; 5 = completely agree)

A response rate of 83% was achieved. Table 2 shows a summary of the responses obtained for the survey, while Figs. 1, 2 and 3 show the distribution of the responses obtained across the different questions. Out of the 29 responses obtained, HCPs agreed that electronic EMs hosted online improve accessibility to the EMs and ensures that they are always referring to the most updated version of the EMs. In addition, HCPs and their patients generally prefer e-RMP over hardcopy EMs (both with a median rating of 4 out of a scale of 5). The results remain largely similar when split into the HCPs’ therapeutic areas, except under Ophthalmology. Ophthalmologists believe that their patients would prefer hardcopy materials over e-RMP (median rating of 1.5 out of a scale of 5), due to concerns that “patients with visual problems may have trouble looking at and reading off a screen”, and that patients may “not [be] able to view e-RMP on devices clearly due to existing vision issues”. These concerns can be addressed by providing alternative e-RMP for ophthalmology patients, such as audio recordings of the EMs. It is also noteworthy that the patients under the ophthalmology group were mainly receiving the product in scope for indications like age-related macular degeneration (AMD) and diabetic macular edema. Based on available literature, AMD usually affects patients above 50 years of age [17]. In comparison, multiple sclerosis which is the indication of the products in scope within the neuroscience group is generally diagnosed at an average age of 31 years in Singapore [18]. Therefore, in addition to concerns of visual impairment, age group of patients could have also resulted in lower rating for e-RMP preference within the responses from the ophthalmology category.

**Table 2:** Summary of survey responses

Survey Question	Rating on a scale of 1–5 (median)* (1 = completely disagree; 5 = completely agree) *n* = number of HCPs
1. “I feel that the electronic educational materials hosted online improve accessibility to the materials e.g. easy to find, will not be misplaced unlike hardcopy materials, assured that I am always referring to the most updated version.”	**Overall: 4** (*n*** = 29)** • Ophthalmology: 3 (*n* = 8) • Neuroscience: 5 (*n* = 20)
2. “I prefer e-RMP over hardcopy materials.”	**Overall: 4** (*n*** = 29)** • Ophthalmology: 2 (*n* = 8) • Neuroscience: 4.5 (*n* = 20)
3. “I believe my patients prefer e-RMP over hardcopy materials.”	**Overall: 4** (*n*** = 29)** • Ophthalmology: 1.5 (*n* = 8) • Neuroscience: 4.5 (*n* = 20)

* ‘Normality test’ plots of the survey responses do not follow a ‘normal distribution’

## Discussion

HAs’ in many countries are shifting their focus towards the digitalization of risk management materials. This is evident from the increasing HA guidances that permit the use of digital materials and/or tools for RMP implementation, either as a primary or secondary approach [19]. For example, the Singapore HA’s post-marketing vigilance guidance was updated on 01 April 2024, where pharmaceutical companies who wish to host RMP EMs on digital platforms are now encouraged to notify HSA [9]. The Ministry of Food and Drug Safety (MFDS) in Korea has also recently updated the Guideline on Risk Management Plan for Medicines in March 2023. One of the updates stated that HCP materials may be implemented fully digital if the rationale is provided to and approved by MFDS [20].

After Singapore, Novartis is currently piloting e-RMP in Australia, Indonesia, Korea, Malaysia and Thailand. Of note, the local HA in Korea has even reached out to Novartis to expand the pilot to certain products [21]. Similar to Singapore’s HA, various HAs like those in Finland, Spain and Germany also host electronic copies of the EMs on their HA websites, serving as a reliable reference for HCPs and/or patients/caregivers to access RMP EMs for locally registered products [22–24].

e-RMP can bring many benefits. Like e-labelling, it is an efficient and sustainable way of communicating critical drug-related safety information to both HCPs and/or patients/caregivers [25]. The QR code card serves as a compact take-home material, informing HCPs and/or patients/caregivers on the RMP EM(s) for the product, while minimizing the need for re-printing with each update. The same QR code would always direct users to the latest EMs hosted online. Therefore, the MAH only needs to upload a copy of the updated EMs onto a website and HCPs and/or patients/caregivers would immediately be able to access the updated materials. Conversely, hardcopy EMs require delivery of the updated materials to each prescribing institution by hand which is highly resource intensive and time-consuming. Furthermore, successful delivery of EMs, particularly patient/caregiver EMs, is highly subject to the time taken by the company representative to deliver the EMs, availability of the HCPs to receive the EMs, as well as the patients’ subsequent visit to the clinic to obtain the EMs. Overall, e-RMP saves resources and more importantly, reduces the time needed for the EMs to eventually reach the target users, allowing for the prompt access to updated safety information about the product.

However, there are also factors that can impact the effectiveness of e-RMP in the real world. Firstly, digital literacy of the target users can affect the overall uptake and acceptability of e-RMP. If users are not comfortable with electronic EMs, they may not refer to the e-RMP. Secondly, e-RMP should be hosted on a platform that is easily accessible to the users. If HCPs or patients/caregivers do not retain the QR code cards, they may have difficulty locating the most updated e-RMP. This may cause them to refer to outdated materials saved offline or print the materials instead, thereby negating the benefits of e-RMP. Thirdly, HCPs must consent to receiving communication from MAHs such as emails regarding updates to the EMs, so that HCPs are periodically reminded of the EMs and their related safety messages. A study reported that continuous education to drive pertinent safety messaging or influence necessary changes in management of treatment may be more effective in improving prescribing behaviors [26]. Most importantly, it is imperative that assessment is performed to determine the feasibility of e-RMP within the local context to account for factors such as local HA requirements which could include, but is not limited to, additional documentation when using digital platforms or validation of the platform.

Local HAs may be hesitant towards permitting implementation of e-RMP due to concerns such as cybersecurity or internet connectivity. Therefore, MAHs or HAs can consider a stepwise approach such as maintaining the physical copies after implementation of e-RMP for an appropriate period of time. This approach has been adopted by HAs for e-labelling, to overcome resistance and enable end-users to be familiar with the concept [8]. This would also provide time for technological advancements that may address the above concerns, allowing HAs to become comfortable with e-RMP as the primary, default format.

While e-RMP may be the first step towards digitalization of aRMMs, this only serves as the starting point to explore the vast potential that comes with leveraging such digital platforms to enhance patient safety. Digitalization of EMs opens up a variety of methods to convey messages more effectively. An example would be to convert how most information is currently presented in RMP EMs such as Portable Document Format (PDF) [27] to one that is more interactive, enhancing user understanding [28]. This includes but is not limited to videos demonstrating the correct method of drug administration, audio recordings for patients with visual impairment, and cartoon clips to simplify explanation to younger patients/caregivers. A structured content format such as Extensible Markup Language can also be utilized for RMP EMs to enable easy searching and integration with other digital platforms.

## Conclusion

e-RMP has the potential to serve as a sustainable and efficient way to provide key safety messages pertaining to a medicine. This article highlights the benefits of digital EMs, not only in terms of cost and time savings to the pharmaceutical company, but more importantly, the value it has created for the HCPs and their patients/caregivers. Significant decrease in turnaround time for the updated EMs to reach the HCPs and their patients/caregivers is essential to educate HCPs and patients/caregivers on any updates in key safety messages of the product on real time basis and ultimately safeguard patient safety. Both regulatory authorities and pharmaceutical companies need to make bold and decisive moves towards digital aRMMs. The authors of this article urge health authorities to look into local regulations, to provide flexibility to allow for e-RMP, and to provide guidance on the requirements for electronic distribution of RMP EMs. Additionally, considering the importance of RMP EMs, there are opportunities to review the data privacy regulations specifically for communicating such safety information to HCPs. Pharmaceutical companies should leverage the increasing digital literacy of the end users of educational aRMMs, and seek to explore alternative formats and platforms for their implementation. Hybrid e-RMP or a gradual roll-out of e-RMP in selected therapeutic areas can be considered to ease into e-RMP.

## Data Availability

No datasets were generated or analysed during the current study.
